# Quercetin Reverses Cardiac Systolic Dysfunction in Mice Fed with a High-Fat Diet: Role of Angiogenesis

**DOI:** 10.1155/2021/8875729

**Published:** 2021-02-19

**Authors:** Shasha Yu, Seo Rin Kim, Kai Jiang, Mikolaj Ogrodnik, Xiang Y. Zhu, Christopher M. Ferguson, Tamara Tchkonia, Amir Lerman, James L. Kirkland, Lilach O. Lerman

**Affiliations:** ^1^Division of Nephrology and Hypertension, Mayo Clinic, Rochester, Minnesota, USA; ^2^Department of Cardiology, First Hospital of China Medical University, Shenyang, Liaoning, China; ^3^Department of Nephrology and Research Institute for Convergence of Biomedical Science and Technology, Pusan National University Yangsan Hospital, Yangsan, Republic of Korea; ^4^Ludwig Boltzmann Institute for Clinical and Experimental Traumatology Donaueschingenstraße 13, A-1200 Vienna, Austria; ^5^Robert and Arlene Kogod Center on Aging, Mayo Clinic, Rochester, Minnesota, USA; ^6^Department of Cardiovascular Medicine, Mayo Clinic, Rochester, Minnesota, USA

## Abstract

Global consumption of high-fat diets (HFD) is associated with an increased incidence of cardiometabolic syndrome and cardiac injury, warranting identification of cardioprotective strategies. Cardioprotective effects of quercetin (Q) have mostly been evaluated in ischemic heart disease models and attributed to senolysis. We hypothesized that Q could alleviate murine cardiac damage caused by HFD by restoring the myocardial microcirculation. C57BL/6J mice were fed standard chow or HFD for 6 months and then treated with Q (50 mg/kg) or vehicle 5-day biweekly for 10 additional weeks. Left ventricular (LV) cardiac function was studied in vivo using magnetic resonance imaging, and intramyocardial fat deposition, microvascular density, oxidative stress, and senescence were analyzed ex vivo. Additionally, direct angiogenic effects of Q were studied in vitro in HUVECs. HFD increased body weight, heart weight, total cholesterol, and triglyceride levels, whereas Q normalized heart weight and triglycerides. LV ejection fraction was lower in HFD vs. control mice (56.20 ± 15.8% vs. 73.38 ± 5.04%, respectively, *P* < 0.05), but improved in HFD + Q mice (67.42 ± 7.50%, *P* < 0.05, vs. HFD). Q also prevented cardiac fat accumulation and reduced HFD-induced cardiac fibrosis, cardiomyocyte hypertrophy, oxidative stress, and vascular rarefaction. Cardiac senescence was not observed in any group. In vitro, ox-LDL reduced HUVEC tube formation activity, which Q effectively improved. Quercetin may directly induce angiogenesis and decrease myocardial oxidative stress, which might account for its cardioprotective effects in the murine HFD-fed murine heart independently from senolytic activity. Furthermore, its beneficial effects might be partly attributed to a decrease in plasma triglycerides and intramyocardial fat deposition.

## 1. Introduction

Noncommunicable diseases, especially cardiometabolic diseases (CMD), have become prevalent over recent decades and are associated with several risk factors [1]. Among them, high-fat diet (HFD) and obesity are considered to be major risk factors for mortality secondary to damage in target organs including the heart, kidney, and liver [2]. HFD increases oxidative stress, reduces mitochondrial oxidative phosphorylation, increases cardiac lipotoxicity, and leads to cardiac hypertrophy, fibrosis, and apoptosis [3, 4], as well as dysregulation of immune response [5]. Furthermore, HFD may also increase microvascular permeability and induce loss of the microcirculation, leading to myocardial dysfunction and interstitial fibrosis [6].

In recent years, cellular senescence has emerged as an essential mechanism of tissue dysfunction in obesity [7]. Cellular senescence, which is often associated with aging, may also be triggered by various stimuli, including DNA damage, cellular stress, telomere shortening, and oncogene activation [8]. Accumulation of senescent cells can cause a premature aging state and mediate cardiovascular remodeling [9]. HFD and obesity can lead to senescent cell accumulation in adipose tissue, pancreas, liver, and kidney associated with insulin resistance, chronic inflammation, oxidative stress, adipokine dysregulation, and vascular impairment [10, 11]. However, whether myocardial cellular senescence is triggered in obesity and contributes to murine cardiac dysfunction remains to be clarified.

Quercetin (Q) and its derivatives possess beneficial properties for the cardiovascular system, including antioxidant, anti-inflammatory, antiapoptotic, and antihypertensive effects [12]. Furthermore, recent studies have identified its ability to eliminate senescent cells [7]. The cardioprotective effects of Q in cardiac injury have been established primarily in experimental models of ischemia-reperfusion injury, with a paucity of studies estimating its effects in CMD [13]. In ApoE-/- hypercholesterolemic mice, oral administration of Q reduced left ventricular (LV) hypertrophy via lipid lowering, and in HFD rats, Q upregulated antioxidant defenses and improved cardiac bioenergetics [14, 15]. Q has been previously shown to be more effective than hesperetin, epicatechin, and other flavonoids in ameliorating the metabolic effects of high-fat-induced adiposity [16], and to have therapeutic potential in heart diseases by reducing obesity, recovering plasma thyroid-hormone levels, and attenuating cardiac oxidative stress [17]. Similarly, Q protects against oxidant-elicited endothelial dysfunction in HFD-fed ApoE(-/-) mice by improving nitric oxide bioavailability [18]. However, the effects of Q on myocardial cellular senescence or microcirculation in HFD have not yet been fully evaluated.

In the present study, we aimed to assess the development of myocardial senescence in murine HFD-induced obesity. We tested the hypothesis that chronic intermittent administration of Q would diminish cardiac remodeling and dysfunction in murine CMD through modulation of myocardial microvascular density and decreased cellular senescence.

## 2. Materials and Methods

**Figure 1:**
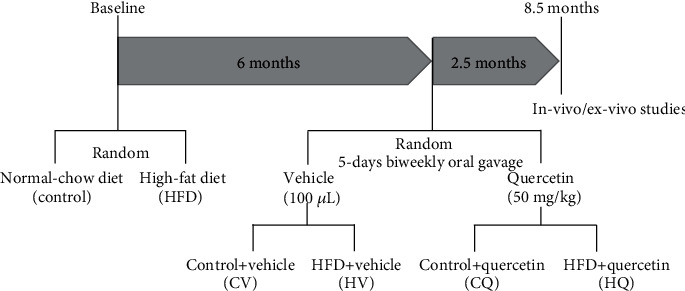
Schematic of the experimental protocol.

For *in vivo* and *ex vivo* studies (Figure 1), four groups of mice were studied (*n* = 6 − 8 each): control+vehicle (CV), control+quercetin (CQ), HFD + vehicle (HV), and HFD + quercetin (HQ). LV cardiac function was evaluated *in vivo* using high-field magnetic resonance imaging, whereas intramyocardial fat deposition, microvascular density, oxidative stress, senescence, and histology were estimated *ex vivo*. *In vitro*, human umbilical vein endothelial cells (HUVECs, Cell Applications, San Diego, CA; Cat.# 200 K-05f) were treated with oxidized low-density lipoprotein (ox-LDL, Thermo Fisher Scientific, Grand Island, NY; Cat.# L34357) to impair capillary generation, and tube formation was assessed to estimate the angiogenic activity of Q.

### 2.1. In Vivo Experimental Model

All animal experiments were approved by the Mayo Clinic Institutional Animal Care and Use Committee. Specific pathogen-free male C57BL/6J mice (Jackson Lab, Bar Harbor, ME) 11 weeks of age were randomly assigned to standard diet (Lab Diet 5053, St. Louis, MO) or HFD (Research Diets, New Brunswick, NJ) (Table 1, Supplementary Table 1). Mice were housed 3-5/cage under standard laboratory conditions: 21-23°C, 50% humidity, 12 h light–dark cycle, municipal city water, and food *ad libitum*. Six months later, all mice were treated with either vehicle (100 *μ*L) or Q (50 mg/kg) 5-day biweekly through oral gavage for ten weeks, based on previous studies [19]. Cardiac function was then assessed using high-field magnetic resonance imaging (MRI) [20]. Mice were euthanized by terminal cardiac blood collection under 2% isoflurane anesthesia, hearts harvested and weighed, and the LV dissected and shock-frozen in liquid nitrogen or preserved in formalin for histology.

### 2.2. Systemic Measurements

Systolic and diastolic blood pressure (BP) and heart rate (HR) were measured shortly before euthanasia by tail-cuff plethysmography and recorded by the CODA™ High Throughput System (Kent Scientific, Torrington, CT). Mice were acclimatized and subjected to handling before measurements, to avoid stress-induced BP elevation during the procedure. BP data were obtained from on average 30 measurements of the same animal at one time. Plasma cholesterol and triglyceride levels were tested at the Mayo Immunochemistry Laboratory using an automatic chemical analyzer. Nonfasting glucose and insulin levels were measures using standard kits (Crystal Chem, Elk-Grove Village, IL; Cat# 81692 and Cat# 90080, respectively), and the homeostasis model assessment of insulin resistance (HOMA-IR) index was calculated as [insulin(*μ*U/l) × glucose(mmol/l)/22.5] [21].

### 2.3. In Vivo Cardiac Function

For assessment of cardiac function, mice were anesthetized with 2% isoflurane and maintained with 1%-2% isoflurane. Warm air was blown onto mice to maintain body temperature at approximately 36°C. Electrocardiogram, respiration, and body temperature were monitored using a physiological monitoring system (SA Instruments, Stony Brook, NY). MRI studies were then performed on a vertical 16.4 T animal scanner equipped with a 38 mm inner diameter birdcage coil (Bruker, Billerica, MA). Images were acquired using a fast-spoiled gradient echo sequence with the following parameters: short-axis slices located from base to apex with slice thickness = 1 mm, field − of − view = 2.56 × 2.56 or 3.0 × 3.0 cm^2^, matrix size = 128 × 128, TR/TE = 154 − 235/2.9 ms, flip angle = 20°, and 15 reconstructed phases per cardiac cycle. We reconstructed and analyzed cardiac cine MRI images off-line using in-house developed software packages in MATLAB (MathWorks, Natick, MA). LV myocardial muscle mass (LVMM), length, diameter, wall thickness, and other cardiac parameters were measured from coronal images. The contours of end-diastolic and end-systolic endocardial and epicardial borders were manually outlined in short-axis images. Papillary muscles were excluded in measurements of LV muscle. LV length was measured using ANALYZE™ (version 12.0, Biomedical Imaging Resource, Mayo Clinic, MN). For LV thickness, we used the minimum value of average thickness of four regions (septum, posterior, lateral, and anterior) in the midlevel LV slice. LVMM was calculated by multiplying end-diastolic LV muscle volume by the specific density of the myocardium. The ejection fraction was calculated as previously described [20]. Stroke volume (SV) was the difference between the maximum and minimum volumes of the LV cavity. Cardiac output (CO) was calculated as SV × HR.

### 2.4. Ex Vivo Cardiac Damages

In paraffin-embedded 5 *μ*m sections, intramyocardial fat deposition was assessed in sections stained with Oil-Red-O (Sigma-Aldrich) and analyzed using ZEN2012 (Carl ZEISS, Germany) [22]. For myocardial fibrosis, trichrome (Newcomer Supply, Middleton, Wisconsin)-stained slides (1 per animal) were examined, and images at ≥10 fields taken at 20× objective. Fibrosis areas were quantified using MATLAB 7.10 (MathWorks) either around (perivascular fibrosis) or excluding (interstitial fibrosis) vessels. Cardiomyocyte cross-sectional area was assessed using both wheat-germ agglutinin (Thermo Fisher, Waltham, MA) and H&E staining. Five fields of each slide were sampled, and the average size of cardiomyocytes was determined using MATLAB. Cardiac capillary density was evaluated from CD31 immunohistochemical staining [23] by quantifying CD31-staining positive areas at ≥10 fields at 10×. Cardiac oxidative stress was determined by cardiac immunoreactivity to 8-hydroxy-2-deoxyguanosine (8-OHdG) (Abcam ab62623, 1 : 100) [7]. The stained sections were observed under fluorescence microscopy, and the density of 8-OHdG staining was analyzed using ImageJ.

Senescence-associated *β*-galactosidase (SA-*β*-Gal) activity and *p16*, *activin-A*, *p21*, and *p53* gene expression were used to estimate myocardial cellular senescence [7, 10, 11]. SA-*β*-Gal activity was studied using a Staining Kit (#9860, Cell Signaling, Boston, MA). Heart tissue sectioned at 10 *μ*m thickness was treated as detailed previously [24]. The degree of senescence (blue color) was quantified in 10 randomly-chosen fields per section using AxioVision (Carl Zeiss SMT, Oberkochen, Germany) and expressed as an average of percent SA-*β*-Gal-positive to total field area. For confirmation, we also used a different SPiDER *β*-Gal staining kit (#1824699-57-1, Dojindo Molecular Technologies, Rockville, MD). Gene expression of the senescence markers *p16*, *activin-A*, *p21*, *p53*, and angiogenic genes, including angiopoietin, vascular endothelial growth factor (VEGF), and its receptor-2 fetal liver kinase- (Flk-) 1, were measured using real-time PCR in cardiac tissues [7]. Relative quantitative PCR (^△△^Ct) was performed using TaqMan assays with primers and probes for *p16* (mm0049449), *activin-A* (mm00434339), *p21* (mm00432448), *p53* (mm01731290), angiopoietin-1 (mm00456503), Flk-1 (mm0122421), VEGF (mm00437306), and GAPDH (mm9999915) loading control (Thermo Fisher Scientific). Negative controls with no cDNA were cycled in parallel with each run. Reactions were run in Applied Biosystems ViiA7 Real-Time PCR systems at 50°C for 2 minutes, 95°C for 10 minutes, 40 cycles of 95°C for 15 seconds, and 60°C for 1 minute. The difference in PCR product yields between the experimental groups was determined by comparing fold changes of each target mRNA after normalization to GAPDH.

### 2.5. In Vitro Studies

To assess the direct effects of Q on capillary formation, HUVECs were grown in endothelial cell growth medium (EGM™-Plus Endothelial Cell Growth Media-Plus BulletKit™ Medium Lonza, Cohasset, MN; Cat.# CC-5035) with or without coincubation with ox-LDL, which reduces their ability to form capillaries [25]. HUVECs were divided in 4 groups [26]: control (culture medium), ox-LDL (100 *μ*g/ml), ox-LDL+10 *μ*M Q (Sigma-Aldrich, St. Louis; Cat.#Q4951), and ox-LDL+20 *μ*M Q. HUVECs were then seeded on a Matrigel® matrix-coated plate (Corning, Corning, NY; Cat.#354433) at a final concentration of 7 × 10^4^ cells/500 *μ*l for 24 h, during which cells were maintained in a 37°C, 5% CO_2_ humidified incubator. For each condition, four independent experiments were performed. HUVECs were observed under a Zeiss Axio Observer inverted microscope for the formation of tube-like structures and networks of tubes counted in five different fields of view for each group and averaged, as previously described [25]. Angiogenesis Analyzer, a plug-in of ImageJ software, was used to estimate the vessel length and branch points for each group.

### 2.6. Statistical Analysis

Statistical analysis was performed using SPSS statistical software (Chicago, IL). The Shapiro-Wilk Test was used to estimate the distribution of the data. Normally-distributed variables are presented using mean ± standard deviation and nonnormal data as median (interquartile range). Statistical significance for parametric variables was determined using one-way analysis of variance (ANOVA) followed by Student's *t*-test and nonparametric variables by Kruskal-Wallis followed by Wilcoxon test. *P* < 0.05 was considered statistically significant.

## 3. Results

### 3.1. Effect of Q on Systemic Parameters in HFD Mice

**Figure 2:**
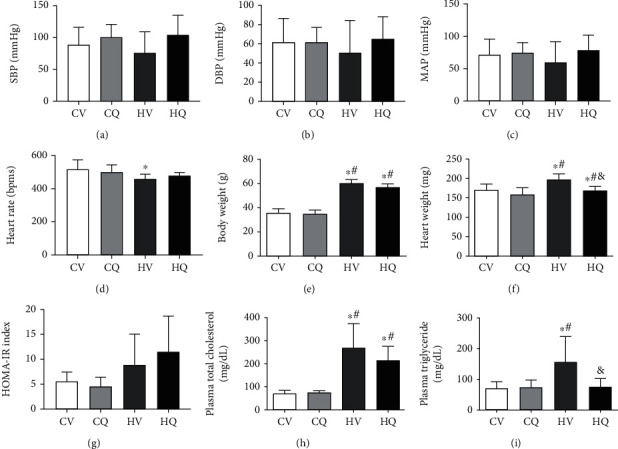
Systemic characteristics (mean ± standard deviation) in C57BL/6J mice. High-fat diet (HFD) increased body and heart weights, plasma total cholesterol, and triglyceride, which quercetin partially alleviated. HFD also tended to induce insulin resistance, reflected in homeostasis model assessment of insulin resistance (HOMA-IR) index. Control+vehicle (CV; *n* = 8); control+quercetin (CQ; *n* = 8); high-fat+vehicle (HV; *n* = 5); high-fat+quercetin (HQ; *n* = 4); ^∗^*P* < 0.05 vs. CV; ^#^*P* < 0.05 vs. CQ; ^&^*P* < 0.05 vs. HV.

Systemic indices of the mice are shown in Figure 2. There were no BP differences among the groups (Figures 2(a)–2(c)), but HFD decreased HR compared to controls (*P* < 0.05) (Figure 2(d)).

HFD significantly increased body weight and heart weight, and Q significantly (*P* = 0.016) decreased heart weight in HFD mice (Figures 2(e) and 2(f)). HOMA-IR showed a trend for increased insulin resistance in both the HV (*P* = 0.188) and HQ (*P* = 0.06) HFD groups, with no effect of Q treatment (Figure 2(g)) but has not reached statistical significance due to variability. HFD mice were also characterized by increased plasma levels of total cholesterol and triglyceride, and Q normalized the latter (*P* = 0.027) (Figures 2(h) and 2(i)). Therefore, treatment with Q reduces obesity-induced increase in heart weight and decreases plasma triglyceride in obese mice.

### 3.2. Effect of Q on Cardiac Remodeling and Dysfunction in HFD Mice

We evaluated the effect of Q on HFD-induced changes in cardiac anatomy and function (Table 2). SV (*P* = 0.002) and CO (*P* = 0.007) were unchanged in HV and CV but increased in HQ compared to HV alone. However, HFD decreased in HV LVEF that was normalized by Q (*P* = 0.026, vs. HV). Thus, Q alleviated cardiac systolic dysfunction in HFD mice.

### 3.3. Effect of Q on Cardiomyocyte Hypertrophy and Myocardial Fibrosis

**Figure 3:**
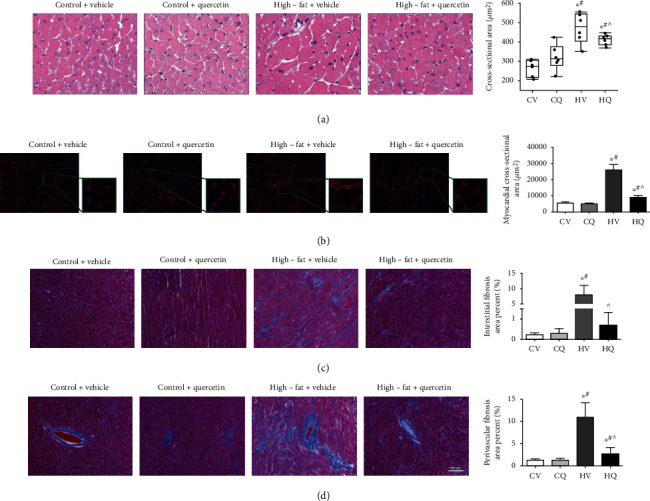
High-fat diet induces cardiomyocyte hypertrophy and cardiac fibrosis in C57BL/6J mice, which quercetin significantly improves. Representative histological sections of hematoxylin-eosin (HE) staining (a) and wheat germ agglutinin (WGA) staining (b) show cardiomyocyte hypertrophy. Control+vehicle (CV; *n* = 6 HE; *n* = 8 WGA), control+quercetin (CQ; *n* = 6 HE; *n* = 6 WGA), high-fat+vehicle (HV; *n* = 6 HE; *n* = 5 WGA), high-fat+quercetin (HQ; *n* = 8 HE; n =6 WGA), and representative histological sections of Masson's Trichrome staining and quantification show increased myocardial perivascular (c) and interstitial (d) fibrosis in high-fat diet-fed mice. CV, *n* = 6; CQ, *n* = 8; HV, *n* = 6; HQ, *n* = 8; Data are mean ± standard deviation. ^∗^*P* < 0.05 vs. CV. ^#^*P* < 0.05 vs. CQ. ^*P* < 0.05 vs. HV.

H&E and WGA staining showed that HFD induced cardiomyocyte hypertrophy, which was improved significantly by Q (Figures 3(a) and 3(b)). Both interstitial (Figure 3(c)) and perivascular (Figure 3(d)) fibrosis were markedly elevated in HV, whereas Q treatment improved perivascular fibrosis and reversed interstitial fibrosis in HQ mice.

### 3.4. Q Alleviated Cardiac Damage in HFD Mice

**Figure 4:**
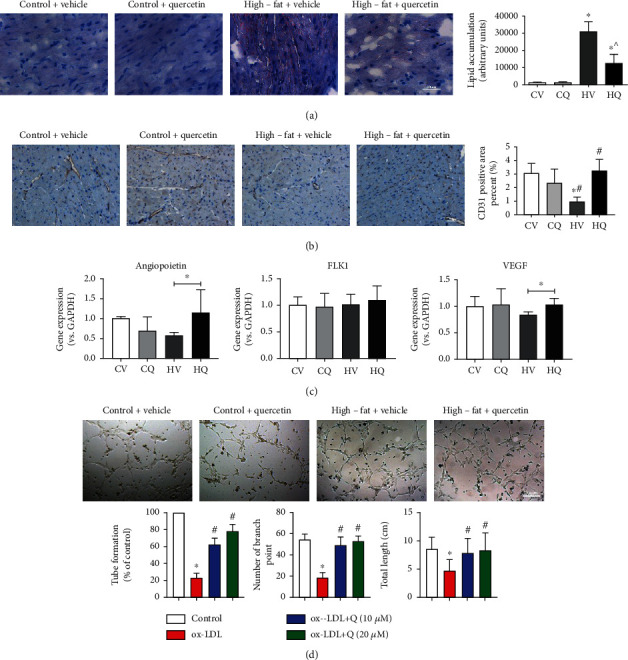
High-fat diet (HFD) elicits lipid accumulation and vascular rarefaction in the myocardium of C57BL/6J mice, which quercetin (Q) alleviated. (a) Accumulation of Oil-red-O-stained lipid droplets in the heart after HFD, which Q blunted. Control+vehicle (CV; *n* = 5); control+quercetin (CQ; *n* = 5); high-fat+vehicle (HV; *n* = 6); high-fat+quercetin (HQ; *n* = 5); (b) Decreased area of CD31 + staining in the HFD heart was improved by Q. Data are mean ± standard deviation. CV, *n* = 7. CQ, *n* = 6. HV, *n* = 5. HQ, *n* = 6. ^∗^*P* < 0.05 vs. CV. ^#^*P* < 0.05 vs. CQ. ^^^*P* < 0.05 vs. HV. (c) Expression of angiopoietin and VEGF increased in the HFD myocardium after Q treatment. CV, *n* = 8. CQ, *n* = 8. HV, *n* = 6. HQ, *n* = 8. ^∗^*P* < 0.05. (d) Effect of oxidized low-density lipoprotein (ox-LDL) and Q on human umbilical vein endothelial cells (HUVEC) tube formation. Representative microphotographs show that Q improved tube formation. ^∗^*P* < 0.05 vs. control; ^#^*P* < 0.05 vs. ox-LDL.

**Figure 5:**
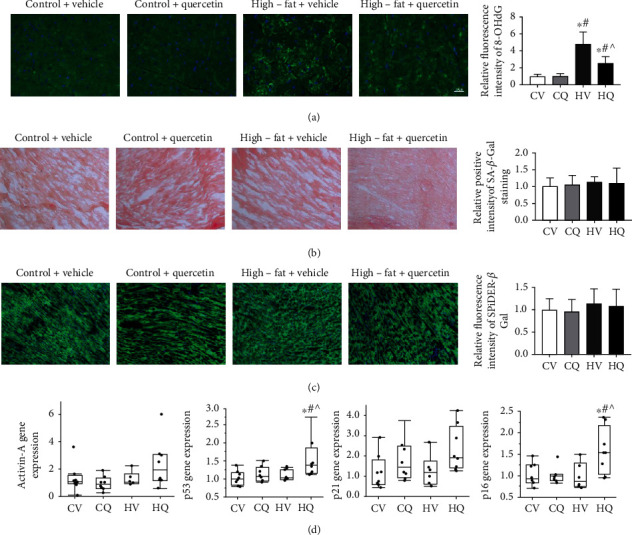
High-fat diet increases oxidative stress but not senescence in the myocardium of C57BL/6J mice; quercetin attenuated myocardial oxidative stress. Data are mean ± standard deviation. (a) Representative immunofluorescence images labels with 8-OHdG in the heart. Control+vehicle (CV; *n* = 7); control+quercetin (CQ; *n* = 6); high-fat+vehicle (HV; *n* = 5); high-fat+quercetin (HQ; *n* = 6). (b) Cardiac colorimetric (blue) staining of senescence-associated *β*-galactosidase (SA-*β*-Gal). CV, *n* = 5. CQ, *n* = 8. HV, *n* = 5. HQ, *n* = 6. (c) Cardiac fluorescent SPiDER-*β*-Gal staining. Neither was different among the groups. CV, *n* = 5. CQ, *n* = 5. HV, *n* = 6. HQ, *n* = 6. (d) Cardiac *p16*, *p21*, *p53*, and Active-A gene expression. Expression of *p16* and *p53* genes was upregulated only in HQ. CV, *n* = 8. CQ, *n* = 8. HV, *n* = 6. HQ, *n* = 8. ^∗^*P* < 0.05 vs. CV. ^#^*P* < 0.05 vs. CQ. ^^^*P* < 0.05 vs. HV.

Accumulation of Oil-Red-O-stained lipid droplets in C57BL/6J mouse hearts increased dramatically in HFD (Figure 4(a)). Interestingly, Q alleviated, although did not fully normalize, lipid myocardial droplet accumulation in HFD. HFD also caused significant myocardial microvascular rarefaction, which Q blunted (Figure 4(b)). Gene expression of angiopoietin (*P* = 0.310) and VEGF (*P* = 0.114) in HV appeared lower yet did not reach a significant difference compared to CV (Figure 4(c)). Yet, in HQ, their expression significantly increased compared with HV. FLK-1 remained unchanged among the groups. In HV, 8-OHdG staining also showed increased oxidative stress in the myocardium (Figure 5(a)), which Q attenuated.

### 3.5. High-Fat Diet-Induced Obesity Does Not Increase Cardiac Markers of Senescence

Myocardial tissue senescence was assessed using colorimetric (Figure 5(b)) and immunofluorescent (Figure 5(c)) SA-*β*-Gal staining, as well as expression of gene related to senescence genes. HV hearts showed no change in SA-*β*-Gal staining or senescent gene expression compared to CV (Figure 5(d)). Interestingly, *p53* and *p16* gene expressions were upregulated in HQ compared to the other three groups.

### 3.6. Q Improves Tube Formation by HUVEC In Vitro

*In vitro*, ox-LDL significantly decreased the number of tubes formed by HUVECs, as well as the number of branching points and their total length. Both doses of Q successfully reversed the ox-LDL-induced angiogenic dysfunction of HUVECs (Figure 4(d)).

## 4. Discussion

Our study shows that murine HFD-induced obesity and dyslipidemia result in cardiac fibrosis, cardiomyocyte hypertrophy, increased intracardiac volume, and impairment of LV systolic function. Chronic intermittent treatment with Q improves cardiac systolic function and attenuates fibrosis and hypertrophy, in association with modulation of intramyocardial fat deposition, myocardial microvascular density, and oxidative stress. Interestingly, these effects were dissociated from senolytic activity, but might be linked to direct proangiogenic properties of Q. These findings suggest that Q could be used to blunt HFD-induced cardiac injury.

Q is a natural flavonol that harbors a variety of biological effects, including antioxidant, anticancer activity, antisenescence, and modulation of angiogenesis [27]. Notably, recent studies position Q as a potent senolytic drug, especially in combination with dasatinib [28]. Intermittent delivery of senolytics targets antiapoptotic mechanisms that defend senescent cells and minimize off-target effects [7]. Indeed, we have previously shown that chronic intermittent Q treatment alleviated renal fibrosis and dysfunction caused by HFD, possibly by attenuating renal cellular senescence [7]. Similarly, in the current study, Q greatly improved HFD-induced perivascular and interstitial fibrosis in the murine heart. However, given the lack of apparent senescence in the HFD heart, this was likely independent of senolytic effects and might have been by direct attenuation of fibrosis in the heart. For instance, Q inhibits rabbit tracheal stenosis by antifibrogenic activity and blunts isoproterenol-induced cardiac ischemia and fibrosis by scavenging reactive oxygen species and suppressing inflammation [29, 30]. In this study, Q upregulated *p*16 and *p*53 myocardial gene expressions in HFD. Pertinently, stimulation of the p53 pathway in cardiac fibroblasts after acute ischemic cardiac injury increases mesenchymal-to-endothelial transition, vascularity, and cardiac function [31]. Possibly, Q might have alleviated cardiac fibrosis, vascular rarefaction, and cardiac dysfunction in HFD partially through stimulation of the *p*53 pathway. Nevertheless, we cannot rule out the possibility that upregulation of *p16* in HQ was not cardioprotective. For example, in hyperlipidemic rats, Q pretreatment before I/R injury increased myocardial infarct size and release of lactate dehydrogenase and creatine kinase-MB [32]. On the other hand, Q upregulated *p*16 expression in colon adenocarcinoma cell lines, causing cell cycle arrest and thereby preventing proliferation of cancer cells [33]. Speculatively, the increase of *p*16 in HQ might arrest fibroblast cells proliferation and thereby decrease cardiac fibrosis. However, further studies are needed to investigate this phenomenon.

In addition, we found that Q decreased oxidative stress and mitigated myocardial vascular rarefaction in the HFD myocardium. Cumulative evidence suggested that diminishing oxidative stress is one of the major cardioprotective mechanisms of Q, which also alleviated myocardial damage in diabetic rats by decreasing inflammation and apoptosis [34]. Moreover, Q shows anticancer effects through its antiangiogenic activity [35]. A previous study showed that at relatively high concentrations, Q inhibited cell viability, Flk-1 expression, and tube formation in a dose-dependent manner (50, 100, 200 *μ*M) in HUVECs [36]. Contrarily, in the murine hindlimb ischemia model, quercetin glucosides enhanced recovery of blood flow to the ischemic leg, manifested by increased capillary density, suggesting its ability to promote angiogenesis in ischemic tissues [37]. Likewise, Q elicited improvement in vessel formation *in vivo* [38]. *In vitro*, a relatively lower concentration of Q (20 *μ*M), as used in the current study, increased HUVEC survival and migration after exposure to high glucose by decreasing oxidative stress [39]. Similarly, Q (5-15 *μ*M) dose-dependently increased HUVEC viability damaged by angiotensin-II [26]. Hence, the angiogenic properties of Q are likely dose- and disease-dependent, akin to statins that can either increase or decrease angiogenesis, depending on the underlying pathology [40]. We used ox-LDL to modulate HUVECs tube formation. Low concentrations of ox-LDL (1-80 *μ*g/ml) promote *in vitro* angiogenesis by endothelial cells, whereas higher concentrations (≥100 *μ*g/ml) impair angiogenesis [25, 41], possibly by oxidative stress. We observed that ox-LDL (100 mg/ml) indeed decreased tube formation ability, which low concentrations of Q reversed.

We have also found that Q reversed myocardial hypertrophy caused by HFD. Tamarixetin, a natural flavonoid derivative of Q with antioxidative and anti-inflammatory properties, also alleviated pressure-overload-induced cardiac hypertrophy in a transverse aortic constriction mouse model [42]. Furthermore, Q improved morphological and functional cardiac parameters in spontaneously hypertensive rats [43]. Importantly, the mice in the current study did not have hypertension, yet HFD-induced myocardial hypertrophy, independent of BP, was also alleviated by Q.

LV fat deposition is also associated with heart diseases such as hypertrophy or acute myocardial infarction, and its mitigation might have mediated the effect of Q on alleviating HFD-induced cardiac hypertrophy [44]. Elevated circulating free fatty acids and triglycerides in HFD cause ectopic myocardial lipid accumulation and impair cardiac systolic and diastolic function [45]. Q has been shown to lower plasma triglyceride levels by increasing their uptake and assembly [46]. Pertinently, Q also improved HFD-induced diastolic dysfunction in rats by preventing cholesterol accumulation and ATP reduction, possibly by regulation of intracellular antioxidant mechanisms and improved cardiac bioenergetics [15]. Our study shows that HFD can also induce in mice systolic dysfunction characterized by decreased ejection fraction that Q normalized, possibly partly by decreasing intramyocardial fat deposition. Furthermore, reversal of cardiac systolic dysfunction by Q might be partially due to its proangiogenic activity, as vascular rarefaction is associated with cardiac dysfunction [47].

Lastly, Q might have reduced systemic cellular senescence in organs other than heart, like visceral fat, kidney, or liver [7, 19]. Improved function in these organs could potentially contribute to the cardioprotective effects of Q. For example, we have shown that selective improvement of kidney function in turn augmented myocardial microvascular function as well [48].

In the present study, we treated HFD mice with intermittent rather than continuous Q dosing. Notably, senolytic drugs can be effective at intermittent administration, because cells can take up to 6 weeks to become fully senescent, nondividing cells [49]. In preclinical models and clinical trials, intermittent treatment with senolytics effectively prevented or alleviated cancers, cardiovascular, liver, kidney, and metabolic disorders [50–52], enhanced cardiac and vascular function in aging mice [52], augmented insulin sensitivity, and reduces adipose tissue inflammation in genetically obese mice [51]. Furthermore, compared with continuous dosing, intermittent administration could reduce off-target and adverse side effects [49]. Evidently, in our study, the intermittent regimen of Q was also efficacious in blunting nonsenescence-related cardiac damage and dysfunction; additional studies are needed to determine whether continuous Q administration bestows more salutary effects.

There are some limitations of the present study. Certain analyses were performed in subgroups due to limited sample availability (see Figure Legends and Methods section). First, previous studies confirmed that HFD could induce dyslipidemia and hyperglycemia, characterized by insulin resistance. Because this was not the focus of our study, plasma samples were not collected under fasting conditions, and glucose and insulin levels were therefore variable. Hence, we used the interplay of glucose and insulin levels by the HOMA-IR index to estimate insulin resistance, which remains a crude estimate. Second, we cannot rule out that some effects of Q were achieved by lipid lowering that prevented, rather than reversed, cardiac injury. Future studies are also needed to establish how the doses used *in vitro* compared to drug levels *in vivo* and the putative mechanisms of the direct cellular effects of Q.

## 5. Conclusions

In conclusion, our study demonstrated that chronic intermittent Q treatment alleviates murine HFD-induced cardiac fibrosis, cardiomyocyte hypertrophy, and LV systolic dysfunction by correcting cardiac vascular rarefaction, suppressing oxidative stress, and decreasing intra-myocardial fat deposition, but not necessarily myocardial senescence. These findings underscore the pathologic effects of HFD on the heart and the cardioprotective effects of Q. Future studies are needed to optimize the regimen and elucidate additional cardioprotective properties of Q.

## Figures and Tables

**Table 1:** Composition of control and high-fat diets used to feed the mice in the study.

By energy (%)	Normal-chow diet (5053)	High-fat diet (D12492)
Protein (% kcal)	23.6	20
Fat (% kcal)	11.9	60
Carbohydrate (% kcal)	64.5	20
Energy density (kcal/g)	4.07	5.21

**Table 2:** Global cardiac structure and function using in vivo Cine MRI in mice.

	Control+vehicle (*n* = 8)	Control+quercetin (*n* = 7)	High-fat+vehicle (*n* = 5)	High-fat+quercetin (*n* = 7)
Wall thickness (mm)	0.85 ± 0.06	0.84 ± 0.10	0.86 ± 0.10	0.86 ± 0.10
LV mass (g)	68.62 ± 6.07	71.06 ± 10.17	72.18 ± 10.90	76.41 ± 12.17
Ejection fraction (%)	73.38 ± 5.04	66.71 ± 3.64	56.20 ± 15.80^∗^^†^	67.42 ± 7.50^‡^
Stroke volume (ml)	40.93 ± 6.10	40.02 ± 5.13	37.40 ± 11.38	49.66 ± 9.66^∗^^†‡^
CO (cm^3^/min)	20.83 ± 2.46	20.04 ± 3.19	17.35 ± 6.04	23.77 ± 4.44^‡^

LV: left ventricle; CO: cardiac output; ^∗^*P* < 0.05 vs. control+vehicle, ^†^*P* < 0.05 vs. control+quercetin, ^‡^*P* < 0.05 vs. high-fat+vehicle.

## Data Availability

The data used to support the findings of this study are included within the article.

## Supplementary Materials

Supplementary Materials Supplementary Table 1: additional ingredients of normal-chow diet and high-fat diet used to feed the mice in the study.
