# Supplementary material for: Quercetin Reverses Cardiac Systolic Dysfunction in Mice Fed with a High-Fat Diet: Role of Angiogenesis
Source: Oxid Med Cell Longev. 2021 Feb 19;2021:8875729. doi: 10.1155/2021/8875729 (PMC7914089; doi:10.1155/2021/8875729)
Supplement: Supplementary Materials — Supplementary Table 1: additional ingredients of normal-chow diet and high-fat diet used to feed the mice in the study. [file 8875729.f1.docx]

| **Supplementary Table 1.** Additional ingredients of normal-chow diet and high fat diet used to feed the mice in the study. | | | |
| --- | --- | --- | --- |
|  | **Normal-chow diet (5053)** |  | **High fat diet (D12492)** |
| ***Protein*** | % |  | gm |
| Arginine | 1.22 | Casein | 200 |
| Cystine | 0.28 | L-Cystine | 3 |
| Glycine | 0.96 | Corn Starch | 0 |
| Histidine | 0.50 | Maltodextrin 10 | 125 |
| Isoleucine | 0.97 | Sucrose | 68.8 |
| Leucine | 1.56 | Cellulose, BW200 | 50 |
| Lysine | 1.16 | Soybean Oil | 25 |
| Methionine | 0.70 | Lard | 245 |
| Phenylalanine | 0.90 | Mineral Mix,S10026 | 10 |
| Tyrosine | 0.59 | DiCalcium Phosphate | 13 |
| Threonine | 0.77 | Calcium Carbonate | 5.5 |
| Tryptophan | 0.26 | Potassium Citrate | 16.5 |
| Valine | 1.00 | Choline Bitartrate | 2 |
| Serine | 1.03 | FD&C Blue Dye #1 | 0.05 |
| Aspartic Acid | 2.19 | Vitamin Mix, V10001 | 10 |
| Gluatmic Acid | 4.34 |  |  |
| Alanine | 1.15 |  |  |
| Proline | 1.47 |  |  |
| Taurine | 0.02 |  |  |
| **Fat**  **(acid hydrolysis)** | % |  |  |
| Cholesterol, ppm | 141 |  |  |
| Linoleic Acid | 2.19 |  |  |
| Linolenic Acid | 0.26 |  |  |
| Arachidonic Acid | <0.01 |  |  |
| Omega-3 Fatty Acid | 0.33 |  |  |
| Total Saturated Fatty Acids | 0.93 |  |  |
| Total Monounsaturated Fatty Acids | 0.99 |  |  |
| **Fiber(Crude)** | **%** |  |  |
| Neutral Detergent Fiber | 16.4 |  |  |
| Acid Detergent Fiber | 6.00 |  |  |
| Nitrogen-Free Extract (by difference) | 52.9 |  |  |
| Starch | 33.9 |  |  |
| Glucose | 0.19 |  |  |
| Fructose | 0.23 |  |  |
| Sucrose | 3.18 |  |  |
| Lactose | 1.34 |  |  |
| **Minerals** | % |  |  |
| **Ash** | 6.1 |  |  |
| Calcium | 0.81 |  |  |
| Phosphorus | 0.63 |  |  |
| Phosphorus(non-phytate) | 0.33 |  |  |
| Potassium | 1.07 |  |  |
| Magnesium | 0.22 |  |  |
| Sulfur | 0.34 |  |  |
| Sodium | 0.30 |  |  |
| Chlorine | 0.51 |  |  |
| Fluorine, ppm | 10 |  |  |
| Iron, ppm | 220 |  |  |
| Zinc, ppm | 87 |  |  |
| Manganese, ppm | 85 |  |  |
| Copper, ppm | 13 |  |  |
| Cobalt, ppm | 0.71 |  |  |
| Iodine, pp, | 0.97 |  |  |
| Chromium, ppm | 0.81 |  |  |
| Selenium, ppm | 0.30 |  |  |
| **Vitamins** |  |  |  |
| Carotene, ppm | 1.5 |  |  |
| Vitamin K,ppm | 3.3 |  |  |
| Thiamin Hydrochloride, ppm | 8.0 |  |  |
| Niacin, ppm | 90 |  |  |
| Pantothenic Acid, ppm | 17 |  |  |
| Choline Chloride, ppm | 2000 |  |  |
| Folic Acid, ppm | 3.0 |  |  |
| Pyridoxine, ppm | 9.6 |  |  |
| Biotin, ppm | 0.30 |  |  |
| B12, mcg/kg | 51 |  |  |
| Vitamin A, IU/gm | 15 |  |  |
| Vitamin D_3_ (added),IU/gm | 2.2 |  |  |
| Vitamin E, IU/kg | 99 |  |  |
| Ascorbic Acid, mg/gm | - |  |  |
